# Supplementary material for: Resting-State Functional Magnetic Resonance Imaging Connectivity Between Semantic and Phonological Regions of Interest May Inform Language Targets in Aphasia
Source: J Speech Lang Hear Res. 2020 Aug 5;63(9):3051–67. doi: 10.1044/2020_JSLHR-19-00117 (PMC7890222; doi:10.1044/2020_JSLHR-19-00117)
Supplement: Supplemental Material S1 [file JSLHR-63-3051-s001.pdf]

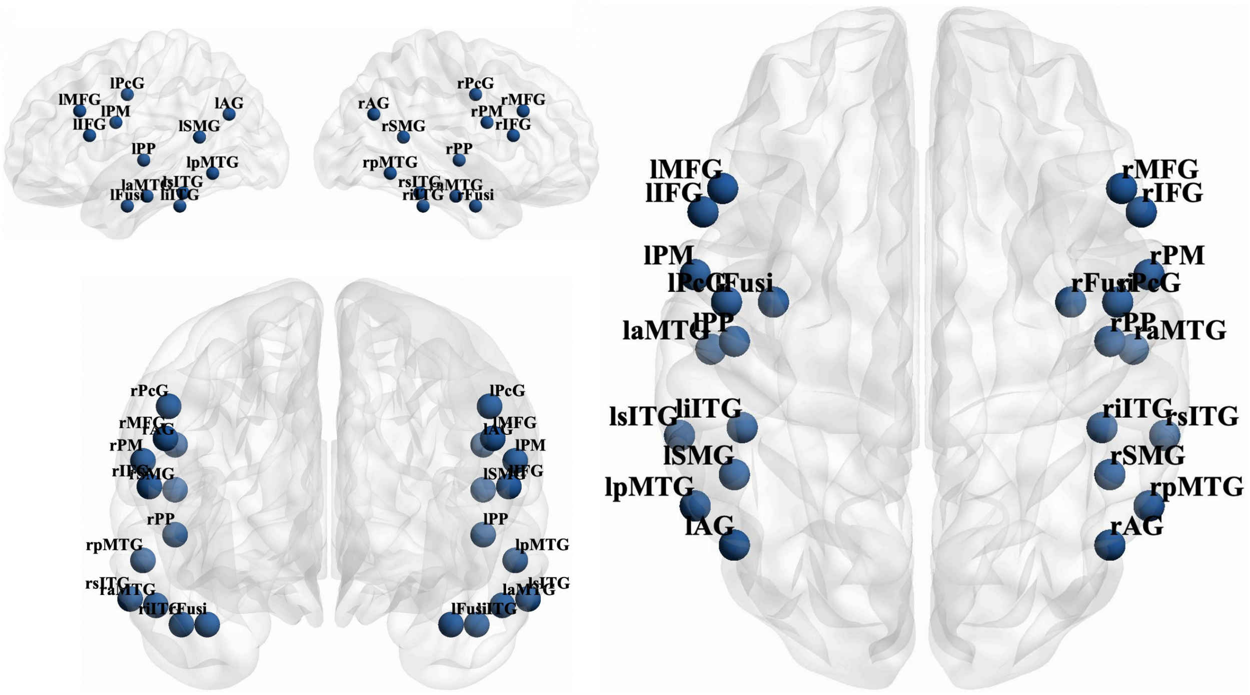

Supplemental Material S1. Region of interest (ROI) maps in the lateral (top left), coronal (bottom left), and axial (right) views.

l: left, r: right, aMTG: anterior middle temporal gyrus, sITG: superior inferior temporal gyrus, iITG: inferior inferior temporal gyrus, Fusi: fusiform gyrus, AG: angular gyrus, PP: planum polare, SMG: supramarginal gyrus, pMTG: posterior middle temporal gyrus, PM: premotor cortex, IFG: inferior frontal gyrus, MFG: middle frontal gyrus.
