# Supplementary material for: Resting-State Functional Magnetic Resonance Imaging Connectivity Between Semantic and Phonological Regions of Interest May Inform Language Targets in Aphasia
Source: J Speech Lang Hear Res. 2020 Aug 5;63(9):3051–67. doi: 10.1044/2020_JSLHR-19-00117 (PMC7890222; doi:10.1044/2020_JSLHR-19-00117)
Supplement: Supplemental Material S2 [file JSLHR-63-3051-s002.pdf]

				Western Aphasia Batter Standard Score						
	Age	Educ	AOS	AQ	SS	Fluency	Content	AC	Rep	N/WF
Age				.09	.04	.01	.10	.11	.15	.02
Sex				.18	.23	.21	.28	.02	.10	.19
Months Post Onset				.05	.01	-.06	.05	.05	.01	-.03
Education				-.11	-.08	-.03	-.15	-.04	-.00	-.19
AOS Severity				-.54**	-.51**	-.61**	-.45*	-.13	-.57**	-.52**
Lesion Volume				-.59**	-.61**	-.52**	-.62**	-.44*	-.35	-.58**
raMTG-laMTG	.28	.31	-.07	.23	.23	.18	.23	.23	.15	.15
raMTG-rSMG	-.11	-.33	.01	-.21	-.27	-.10	-.38	-.19	-.02	-.21
raMTG-riITG	-.51	-.11	.04	-.19	-.22	-.11	-.29	-.02	-.13	-.19
laMTG-latFusi	-.08	-.10	-.14	.18	.21	.06	.32	.01	.07	.26
laMTG-lpSMG	-.21	.20	.25	.07	.04	.05	.02	.31	.07	-.06
latFusi-ratFusi	.34	-.10	-.14	.21	.23	.23	.19	.20	.07	.20
latFusi-lAG	-.23	-.10	-.22	-.20	-.11	-.10	-.09	-.33	-.13	-.20
latFusi-rAG	.02	-.06	-.22	-.03	-.05	-.07	-.02	-.13	.09	-.06
latFusi-riITG	-.38	-.28	-.21	.18	.20	.11	.25	-.26	.20	.29
ratFusi-riITG	-.17	.14	-.28	.18	.24	.15	.28	-.03	.02	.28
lPP-rPP	.39	.08	-.51*	.42	.38	.34	.35	.07	.45	.37
lPP-rSMG	-.10	-.12	-.30	.14	.12	.05	.16	.10	.06	.19
lPP-liITG	-.33	.03	.21	-.14	-.10	-.13	-.05	.09	-.13	-.27
rPP-rsITG	-.32	.23	.21	-.05	-.21	-.17	-.21	.12	.04	.04
lpSMG-rSMG	.02	-.08	-.34	.23	.24	.13	.29	.05	.31	.07

lpSMG-lAG	-.24	.09	.50*	-.34	-.36	-.47*	-.18	.03	-.44*	-.17
lpSMG-lpMTG	.03	.37	-.10	.25	.23	.21	.20	.26	.21	.16
rSMG-rpMTG	-.11	-.11	.06	-.24	-.25	-.29	-.16	-.43	-.07	-.16
rMSG-lsITG	-.20	-.04	.27	-.34	-.17	-.30	-.01	-.32	-.50*	-.17
lAG-rAG	.07	.12	.00	.11	.02	-.03	.06	.38	.15	-.05
lAG-rPcG	.08	-.18	.03	-.01	.02	-.02	.04	.03	-.02	-.05
lAG-lsITG	.09	.01	-.02	.26	.22	.22	.18	.50*	.18	.09
rAG-rPcG	-.23	-.15	.35	-.30	-.33	-.29	-.30	-.14	-.25	-.17
rAG-lsITG	-.01	-.33	-.20	.19	.21	.11	.26	.11	.19	.08
rAG-liITG	-.26	-.30	.16	-.41	-.29	-.24	-.28	-.37	-.46*	-.25
rpMTG-lpMTG	.02	.08	.13	-.06	-.11	-.12	-.07	.19	.02	-.20
rpMTG-rsITG	.03	.11	.10	-.15	-.15	-.25	-.02	.23	-.17	-.25
rpMTG-PM	-.38	-.17	.24	-.02	.07	.02	.10	-.14	-.12	.07
rpMTG-rpIFG	.05	.41	.19	-.39	-.38	-.41	-.28	-.21	-.17	-.49
lpMTG-rsITG	.14	-.03	-.08	.05	-.08	-.13	-.01	.34	.13	-.06
lpMTG-lsITG	-.35	.20	.25	-.21	-.21	-.32	-.06	.22	-.31	-.23
lpMTG-lpIFG	.25	.15	-.04	.16	.12	.13	.09	.19	.26	-.02
rPcG-lPcG	.09	.11	-.43	.24	.28	.23	.28	-.11	.19	.29
rPcG-lPM	.32	-.05	-.31	.12	.22	.20	.20	-.21	.17	.00
rPcG-rPM	.07	.01	-.31	.11	.07	.02	.10	.16	.15	.00
lPcG-lPM	.28	.00	-.13	.33	.31	.39	.17	.05	.30	.33
lPcG-rPM	-.02	.01	-.37	.13	.07	.05	.07	.14	.09	.18
lPcG-lpIFG	.12	.16	.12	.11	.07	.01	.10	.19	.04	.13
lPcG-lMFG	-.09	.16	.03	.02	-.07	-.10	-.03	.12	-.01	.14
rsITG-lsITG	-.02	-.01	.04	.07	.04	.14	-.07	.12	.21	-.13

rsITG-liITG	-.06	.29	-.09	.04	.09	.11	.05	-.03	.05	-.03
rsITG-lMFG	.11	.16	-.06	-.13	-.14	-.34	.08	-.03	-.05	-.20
rsITG-rMFG	-.14	-.01	.04	-.19	-.16	-.28	-.01	-.14	-.22	-.11
lsITG-riITG	.31	.16	-.34	.18	.11	.15	.05	-.08	.30	.17
lsITG-lMFG	.37	.10	-.20	.19	.06	.07	.05	.29	.32	.05
lsITG-rMFG	-.08	-.45*	-.17	.10	.11	.04	.15	-.10	.22	-.02
liITG-riITG	-.37	.15	.23	-.22	-.31	-.16	-.39	.04	-.11	-.22
liITG-lpIFG	.17	.12	.06	.09	.10	.07	.11	-.14	.15	.06
lPM-rPM	.17	-.13	-.54*	.22	.23	.22	.19	.00	.20	.21
lPM-lpIFG	-.39	.11	.53*	-.21	-.14	-.11	-.15	.11	-.44	-.08
rPM-lpIFG	.02	-.07	-.12	.09	.19	.25	.10	.00	-.02	.04
rPM-rpIFG	-.31	.08	-.02	-.03	-.17	-.02	-.28	.27	.03	-.03
lpIFG-rpIFG	.04	-.08	-.39	-.14	-.07	-.03	-.10	-.49*	.06	-.15
lpIFG-lMFG	-.19	.22	.23	-.41	-.37	-.29	-.38	-.30	-.17	-.53*
lpIFG-rMFG	-.12	.12	.16	-.40	-.37	-.30	-.36	-.33	-.30	-.32
rpIFG-rMFG	.02	.07	-.08	-.09	-.01	.09	-.11	-.23	.09	-.28
lMFG-rMFG	-.11	.07	-.14	-.07	-.04	-.05	-.02	-.17	.09	-.21

Supplemental Material S2. Partial correlations controlled for lesion volume, months post onset of stroke, and sex. * $p < .05$, ** $p < .001$.
AOS: apraxia of speech, AQ: aphasia quotient, SS: Spontaneous Speech, AC: Auditory Comprehension, Rep: Repetition, N/WF: Naming and Word Finding.

l: left, r: right, aMTG: anterior middle temporal gyrus, sITG: superior inferior temporal gyrus, iITG: inferior inferior temporal gyrus, Fusi: fusiform gyrus, AG: angular gyrus, PP: planum polare, SMG: supramarginal gyrus, pMTG: posterior middle temporal gyrus, PM: premotor cortex, PcG: precentral gyrus, IFG: inferior frontal gyrus, MFG: middle frontal gyrus.
