# Supplementary material for: Resting-State Functional Magnetic Resonance Imaging Connectivity Between Semantic and Phonological Regions of Interest May Inform Language Targets in Aphasia
Source: J Speech Lang Hear Res. 2020 Aug 5;63(9):3051–67. doi: 10.1044/2020_JSLHR-19-00117 (PMC7890222; doi:10.1044/2020_JSLHR-19-00117)
Supplement: Supplemental Material S3 [file JSLHR-63-3051-s003.pdf]

		PWA		*HC*	
	**Connection**	**Mean**	**SD**	**Mean**	**SD**
Present in Both Groups	lAG-lsITG	.26	.28	.29	.35
	lAG-rAG	.51	.34	.63	.33
	laMTG-lpSMG	.16	.25	.29	.22
	lFusi-rAG	-.10	.17	-.06	.14
	lFusi-rFusi	.13	.24	.18	.20
	liITG-riITG	.40	.27	.53	.29
	lMFG-rMFG	.18	.30	.47	.24
	lPcG-lpIFG	.15	.20	.21	.29
	lPcG-lPM	.32	.31	.34	.26
	lpIFG-lMFG	.21	.23	.25	.24
	lpIFG-rpIFG	.16	.24	.32	.27
	lPM-lpIFG	.24	.26	.13	.17
	lPM-rPM*	.27	.32	.60	.32
	lpMTG-lsITG	.23	.30	.22	.19
	lPP-rPP	.32	.31	.59	.31
	lPP-rSMG	.16	.27	.06	.18
	lpSMG_lAG	.29	.31	.34	.39
	lpSMG_lpMTG	.28	.29	.25	.25
	lpSMG-rSMG	.22	.25	.48	.20
	lsITG-lMFG	.18	.22	.19	.19
	lsITG-riITG	.09	.18	.08	.17
	lsITG-rMFG	.15	.19	.11	.19
	rAG-liITG	-.12	.20	-.17	.20
	rAG-rPcG	-.16	.19	-.10	.20
	raMTG_riITG	.10	.22	.16	.22
	raMTG-rSMG	.20	.21	.19	.24
	rPcG-lPcG	.26	.35	.60	.34
	rPcG-lPM	.28	.32	.38	.37
	rPcG-rPM	.46	.27	.40	.30
	rpIFG-rMFG	.31	.28	.38	.26
	rpMTG-lpMTG	.22	.32	.27	.20
	rpMTG-rpIFG	.16	.24	.15	.26
	rsITG-lMFG	.17	.19	.04	.24
	rsITG-lsITG	.29	.36	.46	.21
	rsITG-rMFG	.27	.29	.09	.24
	rSMG-rpMTG	.27	.26	.25	.19

Present Only in HC	lAG-rPcG	-.10	.21	-.15	.17
	liITG-lpIFG	-.08	.19	-.06	.16
	lPcG-rPM	.16	.27	.33	.22
	lpIFG-rMFG	.09	.23	.29	.29
	lpMTG-lpIFG	.09	.26	.28	.27
	lpMTG-rsITG	.13	.27	.18	.24
	lPP-liITG	-.11	.17	-.13	.14
	rAG_lsITG	.16	.21	.11	.30
	raMTG-laMTG	.31	.27	.53	.25
	rFusi-riITG	.07	.19	.15	.18
	rPM-lpIFG	.08	.19	.09	.12
	rpMTG-lPM	-.11	.24	-.09	.20
	rpMTG-rsITG	.11	.27	.18	.21
	rPP-rsITG	-.11	.17	-.14	.22
	rsITG-liITG	.06	.27	.25	.23
	rSMG_lsITG	-.11	.18	-.15	.22
Present Only in PWA	laMTG-lFusi	.12	.19	.09	.20
	lFusi_riITG	.11	.19	.09	.13
	lFusi-lAG	-.09	.12	-.06	.14
	lPcG-lMFG	.10	.17	.16	.27
	rPM-rpIFG	.10	.23	.09	.17

* HC > PWA, p < .05 (FDR corrected).

Supplemental Material S3. Means and standard deviations (SD) for the connectivity strength of each ROI-ROI connection.

Laterality: r = right, l = left. sITG: superior inferior temporal gyrus, iITG: inferior inferior temporal gyrus, Fusi: fusiform gyrus, AG: angular gyrus, PP: planum polare, SMG: supramarginal gyrus, pMTG: posterior middle temporal gyrus, PM: premotor cortex, IFG: inferior frontal gyrus, MFG: middle frontal gyrus.
