# Supplementary material for: Resting-State Functional Magnetic Resonance Imaging Connectivity Between Semantic and Phonological Regions of Interest May Inform Language Targets in Aphasia
Source: J Speech Lang Hear Res. 2020 Aug 5;63(9):3051–67. doi: 10.1044/2020_JSLHR-19-00117 (PMC7890222; doi:10.1044/2020_JSLHR-19-00117)
Supplement: Supplemental Material S4 [file JSLHR-63-3051-s004.pdf]

Supplemental material, Ramage et al., "Resting-State Functional Magnetic Resonance Imaging Connectivity Between Semantic and Phonological Regions of Interest May Inform Language Targets in Aphasia," *JSLHR*, https://doi.org/10.1044/2020_JSLHR-19-00117

WAB-R Score	Omnibus Test	Laterality	Parameter Estimates			95% Wald CI		Hypothesis Test	
			Parameter	β	Std. Error	Lower	Upper	Wald χ^2	*p*
Aphasia Quotient	$X^2(6, N = 48) = 31.07$, p = .001		(Intercept)	91.51	7.81	76.20	106.83	137.17	.00
AIC = 252			**Lesion Volume**	**-.11**	**.02**	**-.15**	**-.06**	**21.52**	**.00**
BIC = 264			**AOS Severity**	**-5.03**	**1.73**	**-8.43**	**-1.64**	**8.43**	**.01**
	Both groups	B	lPP-rPP	16.37	1.23	-3.67	36.41	2.56	.15
	Both groups	**R**	**rpMTG-rIFG**	**-25.60**	**8.82**	**-42.89**	**-8.31**	**8.42**	**.01**
	Both groups	B	lPM-rPM	-9.36	8.10	-25.24	6.51	1.34	.25
	Both groups	B	lsITG-rMFG	12.88	11.20	-9.09	34.84	1.32	.25
			(Scale)	157.64	4.70	95.04	261.49		
Spontaneous Speech	$X^2(10, N = 48) =$ 36.61, p < .0001		(Intercept)	16.92	2.67	11.68	22.16	4.03	.00
AIC = 166			**Lesion Volume**	**-.03**	**.01**	**-.04**	**-.01**	**12.67**	**.00**
BIC = 183			AOS Severity	-.49	.40	-1.27	.30	1.48	.27
			Gender	.06	1.50	-2.88	2.99	.00	.97
	Both groups	B	lPM-rPM	2.63	2.60	-2.46	7.72	1.03	.34
	Both groups	R	rsITG-rMFG	-4.07	2.57	-9.11	.97	2.51	.21
	PWA only	R	rPM-rIFG	-3.10	1.74	-6.51	.31	3.18	.16
	PWA only	B	lIFG-rMFG	-7.55	4.11	-15.60	.51	3.38	.16
	Both groups	B	lMFG-rMFG	3.93	2.91	-1.78	9.64	1.82	.27
	Both groups	B	lPP-rPP	2.23	1.77	-1.25	5.70	1.58	.27
	Both groups	R	rpMTG-rIFG	-4.36	2.27	-8.80	.09	3.69	.16
			(Scale)	6.68	1.72	4.03	11.08		
Content	$X^2(7, N = 48) = 32.14$, p <.0001		(Intercept)	3.27	2.32	-1.28	7.81	1.98	.21
AIC = 131			**Lesion Volume**	**-.02**	**.01**	**-.03**	**-.01**	**21.78**	**.00**
BIC = 143			**AOS Severity**	**-.77**	**.18**	**-1.12**	**-.43**	**19.43**	**.00**
			**Age**	**.13**	**.04**	**.05**	**.22**	**9.10**	**.01**
	Both groups	B	lPM-rPM	-1.83	.98	-3.75	.10	3.46	.10
	Both groups	R	rsITG-rMFG	.80	1.08	-1.32	2.93	.55	.46
	Both groups	B	lPP-rPP	-1.72	1.39	-4.45	1.00	1.54	.24
	Both groups	**B**	**latFusi-rilTG**	**5.28**	**1.42**	**2.49**	**8.07**	**13.76**	**.00**
			(Scale)	2.50	.65	1.51	4.15		
Fluency	$X^2(10, N = 48) =$ 37.75, p<.0001		(Intercept)	8.22	1.07	6.12	1.32	58.76	.00
AIC = 125			**Lesion Volume**	**-.01**	**.00**	**-.02**	**.00**	**6.56**	**.04**

BIC = 142			AOS Severity	-.22	.25	-.71	.27	.75	.42
			Gender	.30	1.01	-1.69	2.28	.09	.77
	Both groups	B	lPM-rPM	2.20	1.23	-.21	4.61	3.20	.13
	Both groups	L	lpSMG_lAG	-1.25	.88	-2.97	.47	2.04	.21
	Both groups	**B**	**rsITG-lMFG**	**-5.04**	**1.46**	**-7.89**	**-2.18**	**11.92**	**.01**
	Both groups	R	rsITG-rMFG	-2.93	1.27	-5.43	-.44	5.31	.06
	PWA only	R	rPM-rIFG	-1.24	.93	-3.07	.59	1.75	.23
	PWA only	B	lIFG-rMFG	-3.76	1.93	-7.54	.02	3.81	.11
	Both groups	B	lMFG-rMFG	3.19	1.88	-.49	6.87	2.88	.14
			(Scale)	1.72	.44	1.04	2.85		
	$X^2(5, N = 48) = 29.78$,								
Auditory Comprehension	$p < .0001$		(Intercept)	7.10	.63	5.87	8.33	128.08	.00
AIC = 102			Lesion Volume	.00	.00	-.01	.00	1.39	.24
BIC = 111	Both groups	**B**	**lAG-rAG**	**2.16**	**.90**	**.40**	**3.92**	**5.79**	**.03**
	Both groups	**L**	**lAG-lsITG**	**2.19**	**.69**	**.85**	**3.54**	**1.19**	**.00**
	Both groups	R	rpMTG-rIFG	-2.17	1.05	-4.24	-.11	4.25	.06
	PWA only	B	lpMTG-rsITG	1.47	.82	-.13	3.08	3.23	.09
			(Scale)	1.08	.28	.65	1.80		
	$X^2(3, N = 48) = 17.92$,								
Repetition	$p < .0001$		(Intercept)	9.93	.61	8.73	11.13	263.52	.00
AIC = 144			Lesion Volume	-.01	.01	-.02	.01	1.14	.29
BIC = 151			**AOS Severity**	**-.95**	**.23**	**-1.39**	**-.50**	**17.36**	**.00**
	Both groups	R	rsITG-rMFG	-1.84	1.03	-3.87	.18	3.19	.10
			(Scale)	5.03	1.30	3.03	8.35		
	$X^2(5, N = 48) = 33.96$,								
Naming and Word Finding	$p < .0001$		(Intercept)	11.37	.90	9.61	13.14	159.86	.00
AIC = 123			**Lesion Volume**	**-.02**	**.00**	**-.02**	**-.01**	**26.70**	**.00**
BIC = 133			**AOS Severity**	**-.45**	**.17**	**-.79**	**-.11**	**6.64**	**.01**
	Both groups	**R**	**rpMTG-rIFG**	**-3.66**	**1.23**	**-6.08**	**-1.25**	**8.82**	**.00**
	Both groups	**L**	**lIFG-lMFG**	**-4.28**	**.87**	**-5.98**	**-2.58**	**24.31**	**.00**
	Both groups	B	lPM-rPM	-.04	1.10	-2.20	2.12	.00	.97
			(Scale)	2.23	.58	1.35	3.70		

Supplemental Material S4. Generalized linear model results for the first proposed model, based on the use of univariate linear models to identify potential predictors. Significant predictors are in **bold**. Model fit statistics (Akaike's Information Criterion [AIC] and Bayesian Information Criterion [BIC]) are provided for each model. Indicators of whether the functional connection was significant in both groups or the PWA group only, are provided in the column left of each potential predictor connection. The normal distribution produced the best fit model for all analyses.

Supplemental material, Ramage et al., “Resting-State Functional Magnetic Resonance Imaging Connectivity Between Semantic and Phonological Regions of Interest May Inform Language Targets in Aphasia,” *JSLHR*, https://doi.org/10.1044/2020_JSLHR-19-00117

Laterality: B = bilateral, R = right, L = left. AOS: apraxia of speech, l: left, r: right, sITG: superior inferior temporal gyrus, iITG: inferior inferior temporal gyrus, Fusi: fusiform gyrus, AG: angular gyrus, PP: planum polare, SMG: supramarginal gyrus, pMTG: posterior middle temporal gyrus, PM: premotor cortex, IFG: inferior frontal gyrus, MFG: middle frontal gyrus.
