# Supplementary material for: Resting-State Functional Magnetic Resonance Imaging Connectivity Between Semantic and Phonological Regions of Interest May Inform Language Targets in Aphasia
Source: J Speech Lang Hear Res. 2020 Aug 5;63(9):3051–67. doi: 10.1044/2020_JSLHR-19-00117 (PMC7890222; doi:10.1044/2020_JSLHR-19-00117)
Supplement: Supplemental Material S5 [file JSLHR-63-3051-s005.pdf]

Supplemental material, Ramage et al., “Resting-State Functional Magnetic Resonance Imaging Connectivity Between Semantic and Phonological Regions of Interest May Inform Language Targets in Aphasia,” *JSLHR*, https://doi.org/10.1044/2020_JSLHR-19-00117

WAB-R Score	*Omnibus Test*	*Laterality*	*Parameter Estimates*			*95% Wald Confidence Interval*		*Hypothesis Test*	
			Parameter	β	Std. Error	Lower	Upper	Wald ⍰^{2}	*p*
Aphasia Quotient	$X^2(3, N = 48) = 27.87, p < .0001$		(Intercept)	99.55	3.78	92.14	106.96	693.54	.000
	AIC = 250		**Lesion Volume**	**-.14**	**.03**	**-.19**	**-.09**	**26.66**	**.000**
	BIC = 257		**AOS Severity**	**-5.34**	**1.51**	**-8.31**	**-2.38**	**12.45**	**.000**
		**R**	**rpMTG-rIFG**	**-22.64**	**8.42**	**-39.15**	**-6.13**	**7.22**	**.007**
			(Scale)	175.42	45.29	105.75	29.97		
Spontaneous Speech	$X^2(2, N = 48) = 24.38, p < .0001$		(Intercept)	18.98	.97	17.87	2.87	385.17	.000
	AIC = 162		**Lesion Volume**	**-.03**	**.01**	**-.04**	**-.02**	**22.73**	**.000**
	BIC=168		**AOS Severity**	**-1.32**	**.32**	**-1.95**	**-.69**	**16.88**	**.000**
			(Scale)	1.04	2.59	6.05	16.65		
Content	$X^2(4, N = 48) = 28.79, p < .0001$		(Intercept)	3.90	2.31	-.64	8.43	2.83	.092
	AIC = 128		**Lesion Volume**	**-.02**	**.00**	**-.03**	**-.01**	**2.62**	**.000**
	BIC = 136		**AOS Severity**	**-.56**	**.18**	**-.92**	**-.20**	**9.32**	**.002**
			**Age**	**.09**	**.04**	**.03**	**.16**	**7.11**	**.008**
		**B**	**lFusi-riITG**	**3.96**	**1.28**	**1.44**	**6.47**	**9.48**	**.002**
			(Scale)	2.80	.72	1.69	4.64		
Fluency	$X^2(3, N = 48) = 2.91, p < .0001$		(Intercept)	8.78	.59	7.60	9.92	221.11	.000
	AIC = 128		**Lesion Volume**	**-.01**	**.00**	**-.02**	**-.01**	**14.97**	**.000**
	BIC = 135		**AOS Severity**	**-.72**	**.15**	**-1.02**	**-.42**	**22.17**	**.000**
			(Scale)	3.05	.79	1.84	5.07		
Auditory Comprehension	$X^2(2, N = 48) = 19.49, p < .0001$		(Intercept)	6.51	.53	5.46	7.55	149.09	.000
	AIC=106	**B**	**lAG-rAG**	**2.11**	**.77**	**.60**	**3.62**	**7.49**	**.006**

	BIC = 111	**L**	**lAG-lsITG**	**2.75**	**.75**	**1.28**	**4.22**	**13.37**	**.000**
			(Scale)	1.53	.39	.92	2.54		
Repetition	$X^2(2, N = 48) = 16.45, p < .0001$		(Intercept)	9.64	.63	8.41	1.86	236.38	.000
	AIC = 143		Lesion Volume	-.01	.01	-.02	.00	1.88	.171
	BIC = 149		**AOS Severity**	**-.96**	**.24**	**-1.43**	**-.49**	**16.02**	**.000**
			(Scale)	5.29	1.36	3.19	8.77		
Naming and Word Finding	$X^2(4, N = 48) = 33.956, p < .0001$		(Intercept)	11.35	.58	1.21	12.50	379.23	.000
	AIC = 121		**Lesion Volume**	**-.02**	**.00**	**-.02**	**-.01**	**27.55**	**.000**
	BIC = 130		**AOS Severity**	**-.45**	**.15**	**-.73**	**-.16**	**9.05**	**.003**
		**R**	**rpMTG-rIFG**	**-3.66**	**1.22**	**-6.04**	**-1.27**	**9.02**	**.003**
		**L**	**lIFG-lMFG**	**-4.28**	**.87**	**-5.98**	**-2.57**	**24.20**	**.000**
			(Scale)	2.23	.58	1.35	3.70		

* $p < .05$ (FDR corrected)

Supplemental Material S5. Generalized linear model results for the best fit models. Significant predictors are in **bold**. Model fit statistics (Akaike's Information Criterion [AIC] and Bayesian Information Criterion [BIC]) are provided for each model. The normal distribution produced the best fit model for all analyses.

Laterality: B = bilateral, R = right, L = left. AOS: apraxia of speech, l: left, r: right, sITG: superior inferior temporal gyrus, iITG: inferior inferior temporal gyrus, Fusi: fusiform gyrus, AG: angular gyrus, PP: planum polare, SMG: supramarginal gyrus, pMTG: posterior middle temporal gyrus, PM: premotor cortex, IFG: inferior frontal gyrus, MFG: middle frontal gyrus.
